# Fragment-Specific Fixation for Trimalleolar Fractures: Functional and Radiological Outcomes

**DOI:** 10.7759/cureus.72567

**Published:** 2024-10-28

**Authors:** Danuksha K Amarasena, Upamanyu Nath, Abhirun Das, Thomas Collins, Anand Pillai

**Affiliations:** 1 Acute Internal Medicine, University Hospitals of North Midlands, Stoke-on-Trent, GBR; 2 Faculty of Medicine, University of Manchester, Manchester, GBR; 3 Trauma and Orthopaedics, Manchester University NHS Foundation Trust, Manchester, GBR

**Keywords:** ankle fractures, foot and ankle, fragment specific fixation, orthopaedic surgery, trimalleolar fracture, volition

## Abstract

Introduction

A trimalleolar fracture is a complex unstable fracture that usually occurs as the result of rotational injuries of the ankle. Management and understanding of these fractures have evolved greatly over the last decade. Our study aimed to assess the postoperative outcomes following the fixation of these fractures using fragment-specific low profile anatomical fixation implants.

Methods

We retrospectively analyzed patients admitted to our multidisciplinary team unit with a trimalleolar fracture between October 2021 and February 2024. Each fracture was classified using CT imaging and subsequently fixed using fragment-specific implantation (Volition^TM^). In the postoperative period, patients were followed up and assessed functionally and radiologically.

Results

A total of 40 skeletally mature patients were included in this study, each requiring surgical fixation for a tri-malleolar ankle fracture. Patient-reported outcome data collected showed an average Manchester-Oxford Foot Questionnaire (MOXFQ) score of 34.3 (±24.6) and a Foot and Ankle Disability Index (FADI) score of 77.9 (±22.1). All but one fracture successfully achieved radiological union with a mean time to union of 7.4 weeks (5-16 weeks).

Conclusions

Our study is an early demonstration of the promising results that can be observed through the use of fragment-specific low-profile anatomical fixation. Further comparative studies would provide a further understanding of the effectiveness.

## Introduction

The ankle joint is a complex structure that is supported by multiple ligaments which help maintain functional stability. Fractures of the ankle joint are amongst the most frequently encountered fractures accounting for up to 10% of all fractures [1,2]. Fractures involving all three of the medial, lateral, and posterior malleolus are subsequently termed trimalleolar fractures [3]. The posterior malleolus was first described in 1822 and is an anatomical prominence situated on the posterior aspect of the tibial plafond [4].

The management of these fractures largely depends on the stability of the ankle joint post injury. Trimalleolar fractures are often unstable fractures due to the increased likelihood of ligamentous injury. Fractures involving the posterior malleolus often lead to disruption of the origin of the posterior inferior tibiofibular ligament (PITFL). The PITFL extends between the posterior malleolus and the fibula and is integral to syndesmotic stability [5]. Surgical fixation is often required in these fractures to restore stability to the ankle joint following trimalleolar fractures; however, the optimal approach for these complex procedures is yet to be agreed upon.

This study focuses on the functional and radiological outcomes following the use of fragment-specific plating systems (Volition™; Ortho Solutions UK Ltd, Essex, United Kingdom) as a novel approach in the surgical fixation of trimalleolar ankle fractures. The Foot and Ankle Disability Index (FADI) and the Manchester-Oxford Foot Questionnaire (MOXFQ) were used as patient-reported outcome measures (PROMs) in order to determine postoperative functional outcomes in our study.

## Materials and methods

This was a retrospective study that analysed the data of patients who were admitted to Wythenshawe Hospital, Wythenshawe, Manchester, United Kingdom, with trimalleolar ankle fractures between October 2021 and February 2024. The exclusion criteria for our study were those who did not require posterior malleolus fixation, those who required fixation methods other than plate osteosynthesis, and patients who were less than 18 years of age. All patients who fulfilled the inclusion and exclusion criteria were included. The Institutional Clinical Audit Committee Service approved the study (evaluation reference number S300).

Diagnosis and intervention

Anterior-posterior (AP) and lateral radiographic films were utilised as the primary identifying imaging modality for the diagnosis of trimalleolar ankle fractures. We subsequently conducted CT scanning of all patients comprising coronal, sagittal, and axial planes alongside three-dimensional (3D) reconstruction. All fractures were classified using the Mason & Molloy classification [6] and were categorised into subtype 1, 2a, 2b, and 3 fractures. Patients who presented with concomitant ankle dislocation or subluxation that required manipulation were managed by the on-call team, and post-reduction back slab splinting was applied alongside radiographic assessment to evaluate for sufficient reduction quality.

Following the resolution of soft tissue injury, definitive fixation was conducted. In instances of soft tissue injury concern and unstable fractures that could not be maintained in position with a back slab, temporary external fixation was utilised whilst awaiting soft tissue resolution. Definitive fixation was executed using osteosynthesis employing fragment-specific implants (Volition plating system).

Postoperative follow-up

During the postoperative period, patients underwent follow-ups at two, six, 12, and 26 weeks in the outpatient department. Clinical assessment was conducted for evidence of pain, stiffness, infection, nerve damage, complex regional pain syndrome (CRPS), metalwork irritation or failure, and clinical union. AP and mortise radiographs were taken to evaluate the adequacy of fixation, reduction quality, and radiological union. A 12-month postoperative functional evaluation was determined by the use of the MOXFQ and the FADI as PROMs.

The FADI is a 34-point validated questionnaire with each question scored on a five-point Likert scale ranging from 0 to 4 [7,8]. These questions are divided into the Activity subscale, where 0 indicates 'unable to do' and 4 indicates 'no difficulty at all,' and the Pain subscale, where 0 means 'unbearable pain' and 4 means ‘no pain’ [7,8]. Consequently, a higher overall FADI score reflects a better functional capability.

Similarly, the MOXFQ is a 16-point validated questionnaire, also scored on a five-point Likert scale [8]. Each item is rated from 0, indicating the least severity, to 4, indicating the greatest severity. The questions are organized into three subscales: Walking/Standing (seven questions), Social Interaction (four questions), and Pain (five questions) [9]. A lower overall score on the MOXFQ indicates better functional capability. The subscales have demonstrated excellent validity, reliability, and responsiveness.

The 12-month postoperative evaluation was a part of the hospital procedure and radiological data was analysed in retrospect.

## Results

We retrospectively analysed a cohort of 40 patients with trimalleolar fractures who underwent surgical fixation. The cohort comprised 16 male patients and 24 female patients with a mean age of 49 years (range: 20-80). The predominant mechanism of injury was twisting trauma to the ankle, and there was clear right-sided predominance compared to left-sided (29 and 11 cases, respectively). Amongst subtypes of trimalleolar fractures, 20 cases were classified as type 2a, 12 as type 2b, and eight as type 3, all of which were treated using fragment-specific plates (Table 1).

**Table 1:** Demographic data and fracture characteristics Data given as frequency (n) except for age, which is given as mean (range)

Characteristics	Values
Age (years), mean (range)	49 (20-80)
Sex	
Male	16
Female	24
Fracture side	
Right	29
Left	11
Fracture Type	
1	0
2a	20
2b	12
3	8

Of the cohort, two patients developed superficial wound infections both of which resolved following a week of oral antibiotic therapy. None of the patients experienced nerve injury, deep infection, or complex regional pain syndrome. Furthermore, there were no recorded instances of implant failure, impingement, or need for implant removal (Table 2). Radiological union was achieved in all fractures except one, where the medial malleolus fracture component, fixed with cannulated screws, resulted in an asymptomatic fibrous union. The average time to radiological union was 7.4 weeks (range: 5 - 16) (Table 2).

**Table 2:** Clinical outcomes and complication rates following surgical fixation CRPS: complex regional pain syndrome

Outcome Measure	Frequency	Percentage
Clinical Union	40	100%
Radiological Union	39	97.5%
Delayed Union	0	0%
Malunion	2	5%
Non-Union	0	0%
Implant Fracture	0	0%
Implant Loosening / Loss of Fixation	0	0%
Superficial Infection	2	5%
Deep Infection	0	0%
Nerve Injury	1	2.5%
Thrombosis	0	0%
Wound Problems	1	2.5%
CRPS	0	0%
Hardware Removal	0	0%
Time to Union, mean (range)	7.5 (5-16) Weeks	

The functional outcome data was collected at 12 months using PROMs. At the final follow-up, the average MOXFQ index score (Table 3) was 34.3 (± 24.6) and the FADI score (Table 4) was 77.9 (± 22.1).

**Table 3:** Manchester-Oxford Foot Questionnaire (MOXFQ) results

Scores	Pain (n=40)	Walking/Standing (n=40)	Social Interaction (n=40)	Overall MOXFQ Score (n=40)
Average	7.0	10.3	4.4	34.3
Min.	0	0	0	3
Max.	17	27	16	89
Standard Deviation	4.3	8.6	4.3	24.6

**Table 4:** Foot and Ankle Disability Index (FADI) results

Scores	Activity (n=40)	Pain (n=40)	Overall FADI Score (n=40)
Average	65.6	12.3	77.9
Min.	14	4	18
Max.	88	16	103
Standard Deviation	19.5	3.1	22.1

## Discussion

In recent years, there has been a growing interest and discussion regarding the optimal management strategies for trimalleolar ankle fractures, with particular focus on techniques for addressing the posterior component of these complex injuries. Although often underappreciated on conventional x-rays, the increased use of CT scans has led to greater recognition of these injuries and, consequently, a shift in surgical management approaches [10].

Involvement of the posterior malleolus is now recognized as a poor prognostic factor in ankle fracture outcomes [11], largely due to the critical role of the PITFL in maintaining syndesmotic stability [5]. Posterior malleolus fractures can compromise this stability, potentially leading to poor clinical outcomes if not properly managed. Inadequate treatment may result in complications such as checkrein deformities [12-15]. Studies have highlighted significant clinical differences between trimalleolar and unimalleolar fractures [16], with notably better outcomes in patients who underwent fixation of the posterior malleolus compared to those managed non-operatively [17].

Several classification systems describe the posterior fragment of ankle fractures, with the most well-known proposed by Haraguchi et al. [18], Bartoníček et al. [19], and Mason and Molloy [6]. For all our trimalleolar fractures, we used the Mason and Molloy classification to guide management based on fracture configuration.

The literature describes various surgical methods for fixing the posterior malleolus [20,21], with the classical approach involving indirect reduction and AP screws [22-24]. This method may have originated from earlier indications for fixation, which were based on fractures involving more than 25-33% of the articular surface [25,26]. While this approach allows for fracture fixation using AP screws, it has limitations, particularly with comminuted or intraarticular impacted (die punch) fractures. These cases may pose challenges in achieving adequate reduction and compression, especially when securing larger fragments against smaller ones, leading to less stable constructs. Additionally, smaller fragments can be difficult to fix with this method, as larger screws may not fully engage across the fracture site, while smaller screws may not provide sufficient compression [27].

The use of fragment-specific fixation is well-documented for certain types of distal radius fractures [28,29]. In recent years, there has been increasing acceptance and interest in these implants for specific fracture types, particularly when stabilizing specific articular segments is necessary. However, there remains limited literature and evidence regarding the use of fragment-specific fixation in trimalleolar ankle fractures. This study aims to present our experience and initial thoughts on the use of these fragment-specific implants for direct fixation of trimalleolar ankle fractures.

Fracture configuration and surgeon preference play important roles in patient positioning and surgical approach selection for managing these fractures. Where possible, priority was given to addressing the posterior malleolus fracture first, using the appropriate fragment-specific implant. This approach allowed for enhanced radiographic visualization during reduction and minimized interference from other metallic components. Subsequently, lateral malleolus fixation was performed in type 2a fractures, as per the Mason and Molloy classification, while medial malleolus fixation preceded lateral fragment stabilization in type 2b fractures. Fixation of type 3 fractures varied depending on the chosen surgical approach.

Fragment-specific implants are carefully designed and anatomically contoured to address each fragment of trimalleolar fractures individually. When appropriately applied and positioned, these implants provide additional assurance of anatomical reduction, complementing radiological assessments.

In cases of medial malleolus vertical shear fractures or fractures exiting higher up posteromedially requiring plate osteosynthesis, these implants are particularly useful due to their low profile and anatomical contour, reducing the likelihood of metalwork prominence. When properly deployed in posterior malleolus fractures, they cause less irritation to soft tissues and tendons. Hardware-related issues are a major cause of reoperation in these fractures. Jeyaseelan et al. reported a 10% increased risk of complications and a two-fold increase in reoperation rates, primarily due to hardware-related problems [14]. These complications can potentially be avoided with the careful and appropriate use of these low-profile, anatomically contoured fragment-specific implants.

The findings from this study suggest that fragment-specific fixation for foot and ankle fractures provides promising functional outcomes based on patient-reported measures. With a mean MOXFQ score of 34.3 (±24.6), patients demonstrated moderate improvements in pain, mobility, and social function. The MOXFQ is scored on a scale from 0 to 100, where 0 represents the best possible foot health and 100 the worst, making this result indicative of a favourable reduction in symptoms and functional limitations compared to pre-operative expectations. Additionally, a mean FADI score of 77.9 (±22.1) highlights substantial functional recovery, as the FADI has a maximum score of 104 points, where higher scores represent better functionality and minimal disability. Together, these MOXFQ and FADI outcomes underscore the efficacy of fragment-specific fixation in enhancing quality of life and functional independence post-surgery. 

Our study highlights promising results following the use of fragment-specific fixation for trimalleolar ankle fractures; however, it is limited by the lack of uptake of these novel plates and hence the resultant restricted sample size of 40 patients. Furthermore, our study highlights only 12-month functional outcome scores. Functional outcomes can vary considerably with time during the postoperative period. We would hope to continue to develop our findings on larger sample sizes with more frequent measures of functional outcome.

## Conclusions

Ankle fractures are a very prevalent musculoskeletal injury. Fragment-specific fixation can be used in particular cases which require the stabilisation of specific articular segments. Despite the technical difficulties and steep acquisition of skills required for the implementation of these products, fragment-specific plates are extremely valuable to surgeons for broadening their treatment options in the management of complex trimalleolar fracture patterns.

Our study is an early demonstration of the promising results that can be observed through the use of fragment-specific low-profile anatomical fixation. A comparative study comparing functional and radiological outcomes of fragment-specific plates against non-fragment-specific plates would be interesting in further understanding the effectiveness.
